# Chelation-Controlled Oriented and Irreversible Immobilization of Native Antibodies on Photoreactive Magnetic Nanoparticles

**DOI:** 10.1021/acsabm.5c02168

**Published:** 2026-01-28

**Authors:** Yi-Ren Huo, Avijit K. Adak, Sachin K. Kawade, Yi-Ju Chen, Mira Anne C. dela Rosa, Yu-Ju Chen, Chun-Cheng Lin

**Affiliations:** † Department of Chemistry, 34881 National Tsing Hua University, Hsinchu 300044, Taiwan; ‡ Institute of Chemistry, 38017 Academia Sinica, Taipei 115201, Taiwan; § JunZhi Biomedical Co., Ltd., Tainan 744094, Taiwan; ∥ Department of Medicinal and Applied Chemistry, Kaohsiung Medical University, Kaohsiung 807378, Taiwan

**Keywords:** antibody, oriented immobilization, photolabeling, magnetic nanoparticle, bioconjugation

## Abstract

We present a strategy for the irreversible and oriented immobilization of native antibodies (Abs) onto magnetic nanoparticles (MNPs) by integrating Ni^2+^–NTA chelation with diazirine (Dia)-mediated photo-crosslinking. MNPs were co-functionalized with nitrilotriacetic acid (NTA) and photoreactive Dia-2 to create a mixed monolayer NTA/Dia-2@MNPs that selectively binds the His-rich Fc domain of unmodified Abs. Short UV exposure activates Dia-2, generating reactive carbenes that covalently anchor proximal residues and permanently lock the Ab in an oriented configuration. This dual-mode immobilization preserves Fab accessibility, enhances binding performance, and prevents Ab dissociation during stringent washing. We validated the platform using two cancer therapy Abs (trastuzumab and cetuximab) and one cancer biomarker (anti-serum amyloid A, anti-SAA) in cancer cells and human serum. Anti-SAA MNPs fabricated by the NTA-Ni^2+^ method showed a 1.5-fold increase in antigen binding in the serum sample compared to the boronate affinity-based method and a significant (22-fold) improvement over random immobilization. Cetuximab-functionalized oriented MNPs by the current immobilization strategy achieved a 4.7–6-fold enhancement in EGFR pulldown efficiency from human embryonic kidney (HEK293T) and non-small cell lung cancer (NSCLC) models, compared to randomly immobilized controls. Notably, the oriented MNPs enabled co-purification of markedly high interactome coverage of >1000 proteins and differential abundance of downstream proteins. Importantly, this platform requires no prior Ab modification and is compatible with full-length native Abs and stable in complex biological samples (cell or serum). By combining chelation-guided orientation with photoinduced covalent fixation, this strategy addresses key challenges in Ab surface engineering and offers a robust, versatile solution for applications in immunoprecipitation, proteomics, and biomarker discovery.

## Graphical abstract

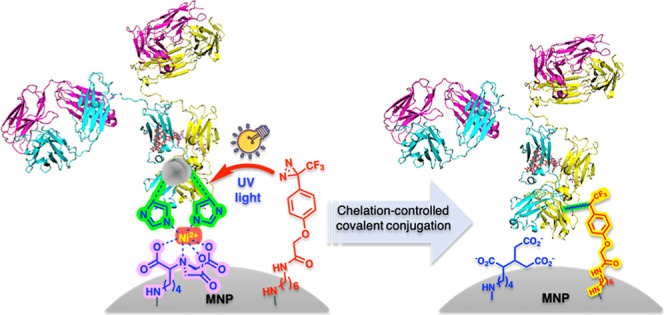

## Introduction

The stable and oriented immobilization of biorecognition elements, such as antibodies (Abs), on solid substrates is essential for the development of a wide range of biomedical and nanobiotechnological devices.^1,2^ For the optimal performance of functional surfaces, it is crucial to achieve a high and well-controlled Ab loading that ensures homogeneous surface coverage and easy accessibility to antigen-binding (Fab) domains. Furthermore, site-selective immobilization strategies that conjugate Abs via the fragment crystallizable (Fc) domain in an irreversible and oriented manner can significantly enhance antigen-binding efficiency and detection sensitivity.^3,4^ To meet these requirements, a variety of immobilization techniques, both non-covalent and covalent, have been developed.^5^ A commonly employed method involves site-specific conjugation via thiol groups generated by reducing the interchain disulfide bonds in the hinge region of Abs, followed by reaction with maleimide-functionalized surfaces through Michael addition.^6^ While this preserves antigen-binding activity, it risks reducing intrachain disulfide bonds and forming unstable maleimide−thiol linkages prone to retro-Michael addition under physiological conditions.^7^ Alternatively, oxidation of *N*-glycans on the Fc domain using periodate generates aldehydes that can react with hydrazides to form hydrazone linkages or be further stabilized by reductive amination.^8^ However, periodate oxidation is chemically harsh and may compromise the structural integrity and activity of the Ab by modifying susceptible residues, such as methionine and cysteine.

Non-covalent strategies utilizing Fc-binding proteins such as Protein A or G^9,10^ have also been widely employed to achieve oriented immobilization. Although effective in improving assay performance, the reversible (non-covalent) nature of the Ab–protein A/G interaction limits their utility in applications requiring strong surface attachment. To address this, engineered Fc-binding proteins, such as Protein Z containing a photoactivatable cross-linker (e.g., *p*-benzoyl-l-phenylalanine), have been developed for covalent immobilization of Ab by photoaffinity.^11^ However, this method relies on recombinant expression and post-translational modification, which adds complexity.

Despite progress in oriented immobilization strategies, achieving site-selective conjugation of full-length native Abs while retaining functional activity remains a considerable challenge.^12^ One promising approach exploits the use of metal chelators, particularly nitrilotriacetic acid (NTA), which can coordinate with nickel­(II) ions for the selective binding of histidine (His)-tagged proteins. Owing to the moderately high affinity of Ni–NTA for poly-His sequences (*K*_D_ ∼ 10^−6^ M),^13^ this system has been extensively used for the site-specific labeling^14,15^ of His-tagged proteins and their oriented immobilization on solid supports.^16^ Notably, the interaction is reversible and can be disrupted by strong chelators such as ethylenediaminetetraacetic acid (EDTA) or excess imidazole, enabling the regeneration of surfaces.

Interestingly, recent studies have suggested that Ni–NTA can target naturally occurring His-rich regions in the Fc domain of IgG Abs, allowing for the potential site-selective immobilization of native, unmodified Abs. Ni–NTA-mediated conjugation has been demonstrated for DNA functionalization near the His-rich cluster in the Fc domain,^17^ and tris–NTA coordination has been employed to chelate Abs to DNA origami structures prior to covalent cross-linking.^18^ Yet, despite its established use in solution-based Ab labeling studies, the application of Ni^2+^–NTA metal affinity interactions for the direct and irreversible immobilization of native Abs onto solid substrates, particularly magnetic nanoparticles (MNPs), remains largely underexplored.^19^

MNPs, particularly superparamagnetic Fe_3_O_4_ nanoparticles, offer a highly versatile platform due to their tunable size, functionalizable surfaces, biocompatibility, and strong magnetic responsiveness.^20^ Ab-functionalized MNPs are increasingly used in bioseparation,^20^ tissue homeostasis,^21^ hyperthermia therapy,^22^ inflammation,^23^ biomarker extraction,^24,25^ and targeting tumor cells^26^ due to their high surface-to-volume ratio and efficient magnetic separability.^8^ However, most existing conjugation strategies rely on the random attachment of Abs, leading to a heterogeneous orientation and substantial loss of bioactivity. To overcome these limitations, site-selective and covalent immobilization approaches have been investigated.^27^ Among them, boronate affinity-based immobilization has shown promise in enabling the self-oriented attachment of native Abs to MNPs via its glycan moieties.^28,29^ Yet, the reversible nature of boronate diester bonds^30^ poses challenges for long-term stability. We have addressed this by incorporating photoactivatable cross-linkers, such as trifluoromethylphenyl diazirine (Dia), which enable covalent tethering of oriented Abs upon UV exposure.^31,32^ We previously reported the successful use of this approach to fabricate stable, self-oriented Ab–MNP conjugates with enhanced capture efficiency.^31^ Furthermore, similar strategies have been used in glycosylated metal nanoparticles for covalent enrichment of lectins.^33^

Herein, we present a new platform based on photoactivatable NTA-functionalized MNPs (NTA/Dia-2@MNPs), designed to achieve irreversible and oriented immobilization of native full-length Abs via coordination with the metal-binding His-rich cluster of the Fc region (Figure 1). Initial validation using trastuzumab, anti-SAA monoclonal Ab, and cetuximab demonstrates that native Abs can be effectively immobilized without compromising antigen recognition. Notably, anti-SAA mAb conjugated to NTA/Dia-2@MNPs showed superior antigen-capture performance from cancer patient serum compared with randomly immobilized Abs. Additionally, we show that cetuximab immobilized via this strategy retains better bioactivity than that when immobilized through boronate-based methods, highlighting the Fc His cluster as a superior site for maintaining antigen-binding functionality.

**Figure 1:**
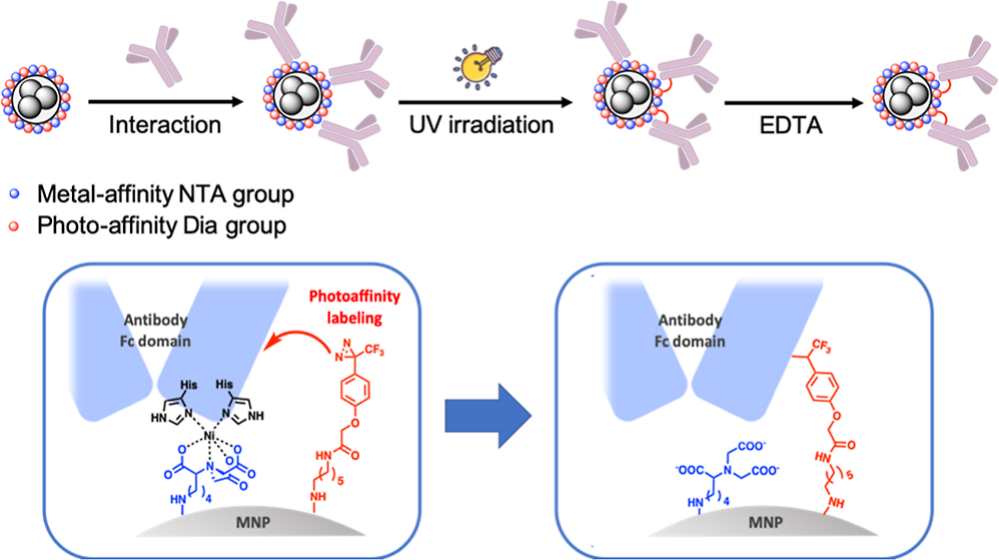
Chelation-controlled, irreversible immobilization of native Abs on photoactivatable NTA-coated MNPs. The Ab initially binds non-covalently through coordination between its Fc metal-binding site and Ni^2+^–NTA on the MNP surface. Upon brief UV exposure, photo-crosslinking occurs, covalently anchoring the Ab via the C_H_3 domain of the Fc region, thereby stabilizing the oriented assembly on the MNP surface.

## Experimental Section

### Fabrication of NTA@MNPs, Dia-1@MNPs, and Dia-2@MNPs

Amine-functionalized magnetic nanoparticles (NH_2_@MNPs) were prepared from FeCl_2_ and FeCl_3_ under basic conditions following our previously reported procedures.^28,31^ Freshly prepared NH_2_@MNPs (1 mg) were washed three times with DMSO and then suspended in a solution of suberic acid bis­(*N*-hydroxysuccinimide ester) (DSS, 5 mg in 100 μL of DMSO). The suspension was shaken at room temperature for 1 h to generate NHS-activated MNPs. After magnetic separation, the particles were washed three times with DMSO. Subsequently, NTA, Dia-1, or Dia-2 (5 mM in DMSO, 100 μL) was added to the activated MNPs, and the reaction mixture was shaken at room temperature for 12 h. The resulting NTA@MNPs, Dia-1@MNPs, or Dia-2@MNPs were magnetically separated and washed three times with DMSO. Residual NHS esters were quenched by incubation with 40 mM 2-(2-(2-methoxyethoxy)­ethoxy)­ethanamine (MEE) in PBS (100 μL) at room temperature for 3 h. After magnetic separation, the particles were washed three times with PBS, resuspended in PBS (100 μL), and stored at 4 °C until use.

### Evaluation of Ab Adsorption on NTA@MNPs, Dia-1@MNPs, and Dia-2@MNPs

MNPs (100 μg) were washed three times with HEPES buffer (20 mM HEPES, 250 mM NaCl, pH 8.0, 0.08% Tween 20) and resuspended in 9 μL of the same buffer. Trastuzumab (10 mg mL^−1^, 10 μL) was pre-incubated with NiSO_4_ or CuSO_4_ (5 mM, 1 μL) for 10 min, followed by addition to the MNP suspension. The mixture was shaken at 4 °C for 10 min. After magnetic separation, Trastuzumab–NTA@MNPs, Trastuzumab–Dia-1@MNPs, and Trastuzumab–Dia-2@MNPs were washed sequentially with PBST (PBS containing 0.05% Tween 20) and PBS (three washes each). The particles were resuspended in PBS (100 μL). Supernatants and MNP pellets were collected for protein analysis.

### EDTA-Induced Dissociation of Trastuzumab–NTA@MNPs

Trastuzumab–NTA@MNPs (1 mg) were magnetically separated and resuspended in PBS (1 mL) containing EDTA at final concentrations of 2.5, 10, and 50 mM. The suspensions were shaken at 4 °C for 10 min. After magnetic separation, the particles were washed three times with PBS. Both supernatants and MNP pellets were collected for protein analysis.

### Fabrication of NTA/Dia-2@MNP

NH_2_@MNPs (1 mg) were washed three times with DMSO and activated with DSS (5 mg in 100 μL of DMSO) by shaking at room temperature for 1 h. After magnetic separation and washing with DMSO, a mixture of NTA (10 mM, 50 μL) and Dia-2 (10 mM, 50 μL) in DMSO was added to the activated MNPs. The reaction mixture was shaken at room temperature for 12 h. Following magnetic separation, the particles were washed three times with DMSO. Unreacted NHS esters were capped with 40 mM MEE in PBS (100 μL) at room temperature for 3 h. The resulting NTA/Dia-2@MNPs were washed three times with PBS, resuspended in PBS (100 μL), and stored at 4 °C.

### Photo-Immobilization of the Antibody on NTA/Dia-2@MNPs

NTA/Dia-2@MNPs (100 μg) were washed three times with HEPES buffer and resuspended in 9 μL of the same buffer. Trastuzumab (10 mg mL^−1^, 10 μL) was pre-incubated with NiSO_4_ (5 mM, 1 μL) for 10 min and added to the MNP suspension. The mixture was shaken at 4 °C for 10 min and then irradiated with UV light (365 nm, 15 mW cm^−2^) at 4 °C for 30 min. After magnetic separation, the particles were washed three times with PBS and incubated with EDTA-containing HEPES buffer (2.5 mM EDTA) at 4 °C for 1 h to remove non-covalently bound Abs. The Ab–NTA/Dia-2@MNPs were washed three times with PBS and resuspended in PBS (100 μL). For fluorescence analysis, Trastuzumab–NTA/Dia-2@MNPs (25 μL) were incubated with Cy3-labeled anti-human IgG (Fab′-specific, 3 μg μL^−1^, 10 μL) at room temperature for 1 h. After being washed with PBST and PBS (three times each), the particles were resuspended in PBS (500 μL), and fluorescence emission was measured (Ex/Em = 550/570 nm).

### Fabrication of BA/Dia-1@MNPs

NH_2_@MNPs (1 mg) were washed with DMSO and activated with DSS (5 mg in 100 μL of DMSO) at room temperature for 1 h. After washing, a solution containing 3-aminophenylboronic acid (5 mM, 50 μL) and Dia-1 (5 mM, 50 μL) in DMSO was added, and the mixture was shaken at room temperature for 12 h. The BA/Dia-1@MNPs were washed with DMSO, capped with 40 mM MEE in PBS for 3 h, washed with PBS, resuspended in PBS (100 μL), and stored at 4 °C.

### Photo-Immobilization of Abs on BA/Dia-1@MNPs (Ab–BA/Dia-1@MNP) and Random Immobilization (R–Ab@MNP) Controls

#### Ab–BA/Dia-1@MNP

BA/Dia-1@MNPs (100 μg) were washed with PBS and resuspended in HEPES buffer (pH 8.5, 9 μL). Ab (10 mg mL^−1^, 10 μL) was added, and the mixture was shaken at 4 °C for 12 h. UV irradiation (365 nm, 15 mW cm^−2^) was performed at 4 °C for 20 min. After washing with PBS, the particles were incubated with dextran (100 μM, 100 μL) at 4 °C for 2 h to remove non-covalently bound Abs. The Ab–BA/Dia-1@MNPs were washed three times with PBS and resuspended in PBS (100 μL).

### R–Ab@MNP

NH_2_@MNPs (100 μg) were activated with DSS (0.5 mg in 10 μL of DMSO) at room temperature for 1 h, washed with DMSO, and incubated with the antibody (2.5 mg mL^−1^, 10 μL) in PBS at 4 °C for 1 h. Residual NHS esters were quenched with 40 mM MEE in PBS, followed by shaking at 4 °C for 12 h. The resulting randomly immobilized Ab@MNPs were washed with PBS and stored at 4 °C.

### Extraction of Human Serum Amyloid A (SAA)

#### Immunoaffinity Purification of SAA

SAA was extracted using a Thermo Fisher KingFisher mL Magnetic Particle Processor. Anti-SAA mAb−NTA/Dia-2@MNPs, anti-SAA mAb−BA/Dia-1@MNPs, or randomly immobilized anti-SAA mAb@MNPs (each containing 2.4 μg of Ab) were incubated with human plasma (5 μL) and diluted with PBS to a final volume of 60 μL at room temperature for 60 min with gentle mixing. Following incubation, the MNPs were separated and washed twice with Tween 20−TBS (20 mM Tris base, 150 mM NaCl, 0.1% Tween 20, TTBS; 100 μL) and twice with ultrapure water (50 μL). The particles were transferred to a fresh microcentrifuge tube, residual liquid was removed, and cytochrome c (internal standard, 5 ng μL^−1^, 1 μL) was added. Subsequently, 2′,5′-dihydroxyacetophenone (2′,5′-DHAP; 2 μL, 10 mg mL^−1^ in 50% ethanol containing 1% TFA) was added as the MALDI matrix. After repeated pipetting to elute bound SAA, the nanoparticles were magnetically separated, and the supernatant was spotted onto a MALDI target plate. After being air-dried, samples were subjected to MALDI-TOF MS analysis.

### MALDI-TOF MS Analysis

MALDI-TOF MS spectra were acquired using a Bruker ultrafleXtreme mass spectrometer (Billerica, MA) equipped with a Smartbeam-II laser, operated in positive reflector mode over a mass range of 5000–20,000 Da. External calibration was performed using a mixture of cytochrome c (12,360.97 Da) and myoglobin (16,952.31 Da). Each spectrum was generated by accumulating 5000 laser shots at 50% laser intensity. Raw spectra were processed by using an in-house modified version of the MALDIquant R package. Baseline subtraction was performed using the SNIP algorithm followed by Savitzky−Golay smoothing (window size = 15). Peaks within the *m*/*z* range of 11,300–11,800 Da were extracted using a signal-to-noise ratio threshold of 10. The intensity of the internal standard (ISD, cytochrome c, *m*/*z* 12,360) was also extracted for normalization. The normalized intensities of all features were summed to obtain the total SAA/ISD, which was used to compare different MNP batches.

### Extraction of Human Epidermal Growth Factor Receptor (EGFR)

#### Cell Culture

Two non-small cell lung cancer (NSCLC) cell lines were used: CL68 cells harboring EGFR exon 19 deletion and T790 M mutation and PC9 cells harboring an EGFR exon 19 deletion. Cells were cultured in RPMI-1640 medium supplemented with 10% fetal bovine serum and 1% antibiotic−antimycotic at 37 °C in a humidified atmosphere containing 5% CO_2_.

### Protein Extraction

Cells (∼1 × 10^7^) were harvested from a 15 cm dish, washed three times with PBS, scraped into ice-cold PBS, and resuspended in ice-cold lysis buffer (150 mM NaCl, 100 mM sodium phosphate, pH 7.2, 1% NP-40, 10% glycerol) supplemented with a protease inhibitor cocktail (100:1, v/v). Cell lysates were sonicated on ice for 5−10 min and collected by centrifugation at 13,000 rpm for 20 min at 4 °C. Protein concentrations were determined using a BCA assay.

### Immunoprecipitation of the EGFR Protein

Cetuximab-conjugated MNPs (25 μg), prepared via NH_2_-, BA-, or NTA-based immobilization strategies, were used to purify endogenous EGFR from 2.5 mg of total cell lysate. Prior to use, Ab@MNPs were washed twice with lysis buffer. Immunoprecipitation was performed by incubating the Ab@MNPs with cell lysates at 25 °C for 1 h with shaking at 800 rpm. Following incubation, the particles were washed sequentially with lysis buffer (1 mL), TBS containing 0.5% Tween 20 (1 mL), TBS (1 mL), and ultrapure water (1 mL). Bound proteins were eluted with 50 μL of TBS containing 1% SDS by shaking at 1000 rpm for 15 min at 25 °C. The nanoparticles were magnetically separated, and the eluates were collected into a 1.5 mL Eppendorf tube for the following experiments.

### Gel-Assisted Digestion

Eluted proteins were subjected to gel-assisted digestion, with minor modifications. Briefly, proteins were polymerized directly in microcentrifuge tubes by adding 17.8 μL of 40% acrylamide/bis­(acrylamide) (29:1), 2.5 μL of 10% ammonium persulfate, and 1.07 μL of TEMED. The resulting gels were cut into small pieces and washed repeatedly with 25 mM triethylammonium bicarbonate (TEABC) containing 50% acetonitrile to remove SDS. Gel pieces were dehydrated with 100% acetonitrile and dried by a SpeedVac. The dried gels were resuspended by 25 mM TEABC, and proteins were reduced with 5 mM TCEP at 37 °C for 30 min and alkylated with 10 mM iodoacetamide at 37 °C for 1 h in the dark. After additional washing and dehydration steps, the gels were rehydrated in 25 mM TEABC and digested with trypsin (protein/trypsin = 20:1, w/w) overnight at 37 °C. Peptides were extracted from the gel using 200 μL of 0.1% (v/v) TFA in 50% CAN twice and 200 μL of 100% ACN, combined, and concentrated by a SpeedVac. The digested peptides were desalted using reversed-phase SDB-XC (3M, USA) StageTips, dried, and stored at − 30 °C until LC–MS/MS analysis.

### LC–MS/MS Analysis

NanoLC−nanoESI−MS/MS analysis was performed using an EASY-nLC 1200 system coupled to an Orbitrap Fusion Lumos mass spectrometer (Thermo Fisher Scientific, Bremen, Germany) equipped with a Nanospray Flex ion source (Thermo Fisher Scientific, Bremen, Germany). Peptides were separated on a 75 μm × 25 cm PepMap C18 column (Thermo Fisher Scientific) (2 μm particles, 100 Å pore size) using a 120 min segmented gradient, with 5−35% solvent B (80% acetonitrile, 0.1% formic acid) over 90 min at a flow rate of 300 nL min^−1^. Solvent A consisted of 0.1% formic acid in water. The mass spectrometer was operated in the data-dependent mode. Briefly, the survey scans of peptide precursors from 350 to 1600 *m*/*z* were performed at 120 K resolution with a 2 × 10^5^ ion count target. Tandem MS was performed by the isolation window at 1.6 Da with the quadrupole, HCD fragmentation with a normalized collision energy of 30, and MS2 scan analysis at 30K resolution in the orbitrap. The MS2 ion count target was set to 5 × 10^4^, and the max injection time was 54 ms. Only those precursors with charge states 2 and 6 were sampled for MS2. The instrument was run in top speed mode with 3 s cycles; the dynamic exclusion duration was set to 15 s with a 10 ppm tolerance around the selected precursor and its isotopes, and monoisotopic precursor selection was enabled.

### Data Analysis

Raw LC−MS/MS data were processed using Proteome Discoverer 2.5 (PD2.5; Thermo Fisher Scientific) with the SEQUEST search engine against the SwissProt human database (v2022_01, 20,307 entries) with a percolator (strict false discovery rate (FDR) of 0.01 and a relaxed FDR of 0.05). Trypsin was specified as the protease with up to two missed cleavages. Mass tolerances were set to 10 ppm for precursor ions and 0.05 Da for fragment ions. Variable modifications included methionine oxidation, cysteine carbamidomethylation, asparagine/glutamine deamidation, N-terminal acetylation, and serine/threonine/tyrosine phosphorylation. Peptides were considered to be identified if their individual ion score was higher than the identity score (*p* < 0.05). To evaluate the false discovery rate (<1%) in protein identification, a decoy database search against a randomized decoy database created by PD2.5 using identical search parameters and validation criteria was also performed. Peptide-spectrum matches (PSMs) with at least high confidence and a strict maximum parsimony principle (target FDR < 0.01) were applied for protein levels. Label-free protein quantification was performed based on precursor ion areas with retention time alignment within 10 min. Quantified proteins were further analyzed using Ingenuity Pathway Analysis (IPA) to evaluate protein−protein interactions and associated signaling networks.

## Results and Discussion

### Magnetic Nanoparticles Design, Fabrication, and Characterization

Figure 1 illustrates the concept of fabrication of covalent and oriented Ab-coated MNPs. We functionalized MNP surfaces with NTAs and photoreactive Dia groups. Incubation of Abs with NTA/Dia-2@MNPs, Ni^2+^-mediated chelation occurred between the NTA ligand on the MNP surface and the His cluster presented at the Fc domain of the Ab. Upon UV irradiation, the Dia group generated carbene that was inserted into the proximal heteroatom–hydrogen bond (e.g., O–H or N–H), forming covalent linkages. Post-irradiation treatment with EDTA removed non-covalently bound Abs, yielding site-selective, irreversibly conjugated immuno-MNPs.

Amine-functionalized MNPs (NH_2_@MNPs) were prepared as described previously in refs 28 and 31 . Briefly, magnetic Fe_3_O_4_ nanoparticles were first coated with a silica shell using tetraethyl orthosilicate (TEOS), followed by a silanization process with (3-aminopropyl)­triethoxysilane (APTES) to introduce amine groups on the nanoparticle surfaces. Then, as shown in Scheme 1, NH_2_@MNPs were first activated using disuccinimidyl suberate (DSS) to form NHS@MNPs. These were subsequently conjugated with lysine (Lys)-derived NTA ligands, *N*_α_,*N*_α_-bis­(caboxymethyl)­Lys (**1**)^34^ for metal-chelate-based binding (NTA@MNPs), and aliphatic Dia linkers (**2**)^31^ (Dia-1@MNPs), or aryl trifluoromethyl Dia linkers (**3**)^32^ (Dia-2@MNPs) for covalent, light-activated photoaffinity labeling. Excess NHS esters on the surface were quenched with 2-(2-(2-methoxyethoxy)­ethoxy)­ethanamine (MEE, 40 mM) prior to Ab immobilization. The preparation and evaluation of these MNPs enable a comparable analysis of distinct surface functionalities that can influence full-length Ab immobilization via photoaffinity labeling. NTA@MNPs served as a control platform for site-directed, metal-chelate-mediated capture, providing a baseline for specific interaction in the His-rich region. In contrast, Dia-functionalized MNPs, Dia-1@MNPs, and Dia-2@MNPs offer covalent photolabeling capabilities through aliphatic and aryl Dia moieties, which differ in reactivity, insertion preference, and labeling efficiency. This strategic variation allows systematic evaluation of the immobilization yield, orientation, and nonspecific binding under photochemical conditions, ultimately guiding the optimal design for robust, covalent Ab conjugation to MNPs.

**Scheme 1:**
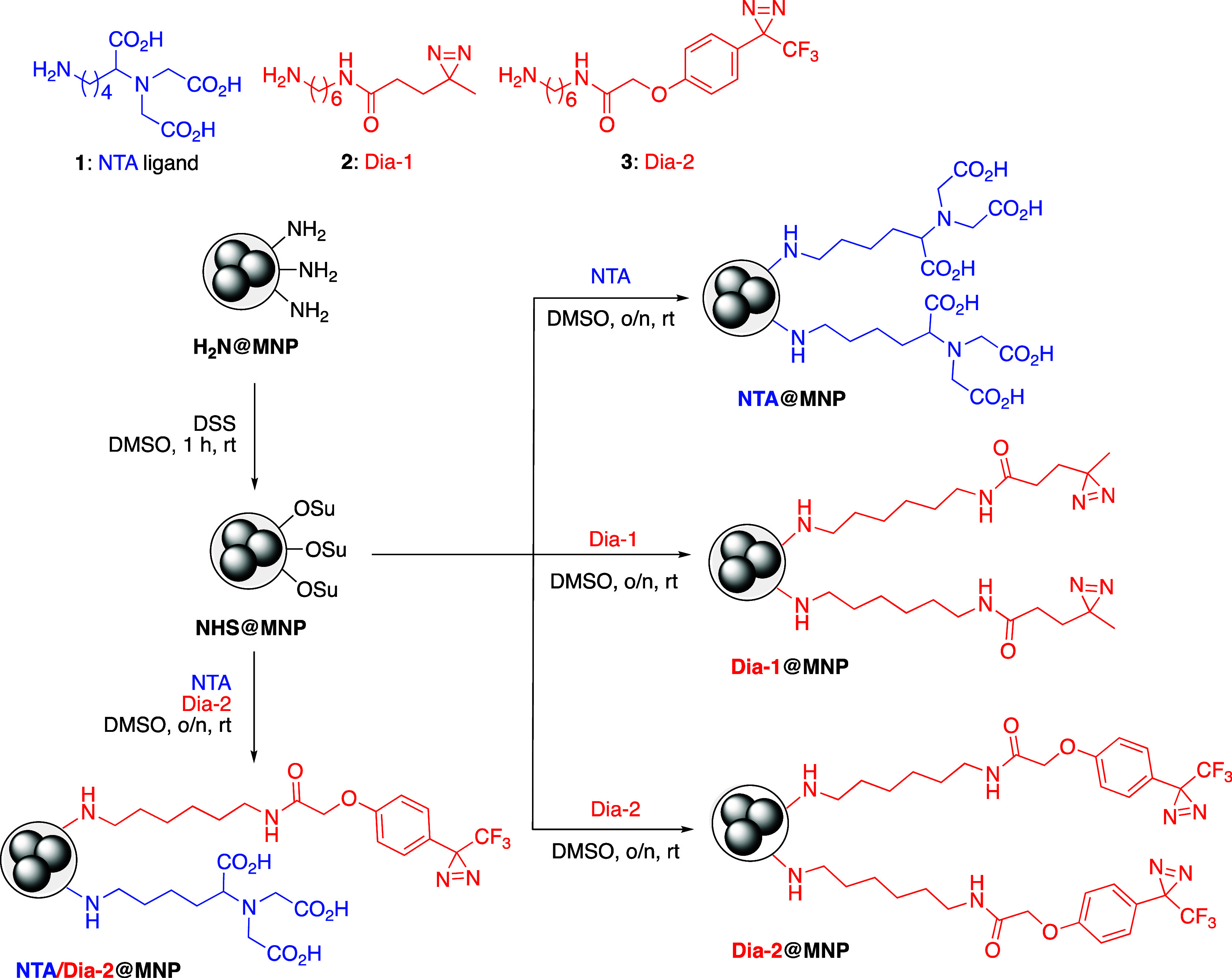
Fabrication of Surface-Functionalized MNPs: NH_2_@MNPs Were Converted to NHS@MNPs and Subsequently Modified to Yield NTA@MNPs, Dia-1@MNPs, Dia-2@MNPs, and NTA/Dia-2@MNPs. Structures of the NTA Ligand (1) and Dias (2 and 3) Used to Prepare MNPs Are Also Shown at the Top Panel

TEM analysis showed that the Fe_3_O_4_ nanocore comprised clusters of 12 ± 3 nm nanoparticles (Figure S1). After sol–gel coating with TEOS and APTES, the resulting NH_2_@MNPs showed an average size of 44 ± 2 nm, with NTA@MNPs (45 ± 3 nm), Dia-1@MNPs (43 ± 3 nm), and Dia-2@MNPs (46 ± 4 nm) (Figure S1c–e), showing no major changes, indicating that the chemical modification only occurred at the outer layer of the nanoparticle surface. Zeta potential analysis revealed a negatively charged NTA@MNPs surface (−17.05 ± 1.67 mV), while Dia-1@MNPs (2.27 ± 0.68 mV), Dia-2@MNPs (3.39 ± 1.43 mV), and NH_2_@MNPs (1.73 ± 0.82 mV) were slightly positive or neutral.

### Metal-Dependent Binding

To demonstrate metal-dependent Ab binding, Trastuzumab (Herceptin) was used as a model monoclonal Ab since it is a well-known therapeutic agent for HER2-positive breast cancer treatment.^35^ NTA@MNPs (100 μg) charged with Ni^2+^ or Cu^2+^ (5 mM) were incubated with Trastuzumab (10 μg). SDS-PAGE showed complete capture of Ab in the presence of metal ions (Figure 2a, lanes 3 and 5), while uncharged NTA@MNPs showed no binding (lane 7). NH_2_@MNPs with Ni^2+^ also failed to bind Ab (lane 8 vs 9), proving specificity. EDTA treatment (2.5–50 mM) efficiently released captured Ab, confirming the reversibility of Ni–NTA interaction (Figure S2). The possibility of its rebinding to the Abs remaining in the supernatant was minimal in the presence of a high-concentration EDTA solution. In addition, the incubation time could be as short as 10 min and tolerated a mild pH range (pH 6–9) (Figure S3).

**Figure 2:**
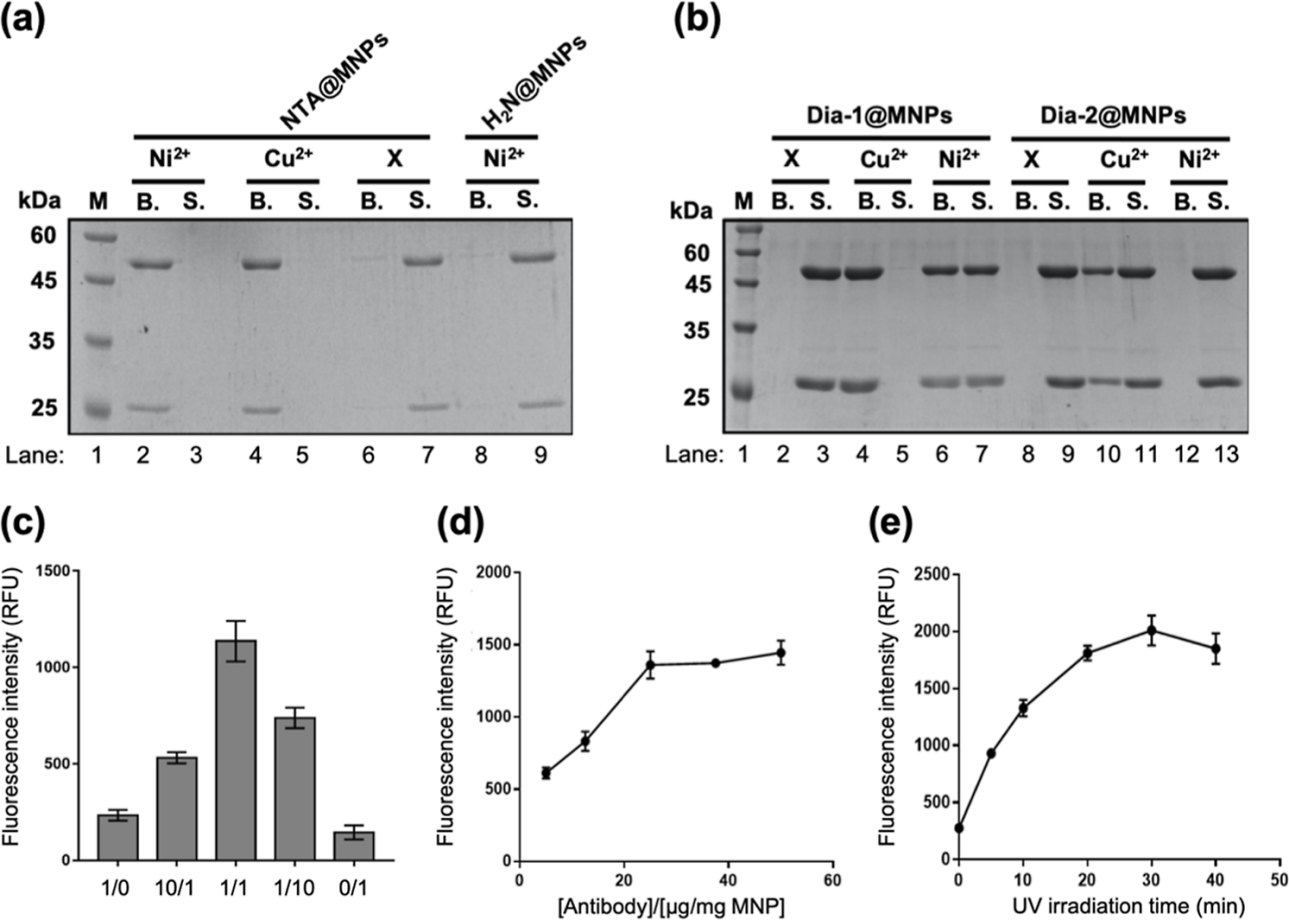
SDS-PAGE analysis of (a) Trastuzumab binding to NTA@MNPs with or without Ni^2+^ or Cu^2+^ and to NH_2_@MNPs with Ni^2+^; (b) nonspecific Ab adsorption to Dia-1@MNPs and Dia-2@MNPs in the presence or absence of divalent metal ions (Ni^2+^ or Cu^2+^). Lane 1: protein marker (M); B: nanoparticle-bound fraction; S: supernatant. Bands at ∼50 and ∼25 kDa correspond to the Trastuzumab heavy chain (HC) and light chain (LC), respectively. (c) Fluorescence intensity of Cy3-anti-IgG Ab binding to Trastuzumab immobilized on NTA/Dia-2@MNPs with varying NTA/Dia-2 ratios with UV irradiation. Optimization of Ab immobilization on NTA/Dia-2@MNPs: (d) effect of Ab concentration and (e) effect of UV irradiation time. All experiments were performed with 365 nm UV light at 4 °C. Error bars (in c–e) represent standard deviation from triplicate experiments.

### Evaluation of Nonspecific Binding

Dia-1@MNPs and Dia-2@MNPs were evaluated for nonspecific Ab binding in the presence of Ni^2+^ or Cu^2+^ (Figure 2b). In the absence of metal ions, neither MNPs showed Ab binding (lanes 2 and 8). Upon the addition of metal ions, Ni^2+^ induced moderate Ab adsorption to Dia-1@MNPs (lane 6) but not to Dia-2@MNPs (lane 12). In contrast, Cu^2+^ showed substantially stronger absorption to both MNPs, with complete depletion of Ab from the supernatant for Dia-1@MNPs (lane 4 vs lane 5), indicating a higher affinity than Ni^2+^ (lanes 4 and 5 vs lanes 6 and 7). A similar trend was observed for Dia-2@MNPs, though the extent of binding was relatively lower compared to Dia-1@MNPs (lane 10 vs lane 4). Notably, Dia-2 presented reduced Ab binding in the presence of a metal cation than Dia-1, likely due to the electron-withdrawing trifluoromethylphenyl group weakening its coordination with cations. Nevertheless, Cu^2+^ is well-known for its strong coordination with many nitrogen-based donor ligands,^36^ and Cu^2+^ is a related electron-rich species compared to Ni^2+^. Thus, Dia-2 could still form a complex with Cu^2+^.

Pleasingly, Dia-2@MNPs showed negligible nonspecific Ab binding in the presence of a Ni^2+^ ion (lane 12), confirming its selective interaction with Cu^2+^. Importantly, SDS-PAGE analysis of the Cu^2+^/Ab/Dia-1@MNP complex before and after photoirradiation showed no apparent change in the band intensities of the heavy and light chains (Figure S4), suggesting very little or no covalent crosslinking of Abs to the nanoparticle surfaces. This observation was further verified by BCA assay results (data not shown). This is possible because the Dia moiety was also considered to act as a co-ligand in forming metal coordination complexes,^37^ thereby suppressing photolabeling efficiency. Based on the minimal nonspecific binding observed and the reduced photolabeling reactivity, Ni^2+^ and Dia-2 were selected as the optimized pair for subsequent studies.

### Oriented Ab Immobilization on the NTA/Dia-2@MNPs Surface

To achieve oriented and irreversible immobilization of Abs on MNPs, we fabricated a mixed monolayer NTA/Dia-2@MNPs surface (Scheme 1). Since both the density of the affinity ligand (NTA) and the photoreactive Dia (Dia-2) would influence the efficiency of Ab binding and covalent cross-linking, we systematically investigated five different molecular ratios of NTA to Dia-2 (1/0, 10/1, 1/1, 1/10, and 0/1). NHS-activated MNPs (NHS@MNPs) were reacted with these NTA/Dia-2 mixtures, and the resulting particles were capped with MEE to quench unreacted NHS esters. The particles were then characterized by transmission electron microscopy (TEM) (see below). To test Ab immobilization, a fixed amount of Trastuzumab (10 μg) was incubated with each formulation of NTA/Dia-2@MNPs (0.1 mg) in the presence of 0.5 mM Ni^2+^ in HEPES buffer (20 mM HEPES, 250 mM NaCl, 0.02% tween 20, pH 8) at 4 °C for 10 min. Following magnetic separation to remove unbound Abs, the MNP complexes were subjected to UV irradiation at 365 nm for 20 min on ice (PBS, pH 7.4), enabling photo-crosslinking through Dia-2 activation. To eliminate any remaining non-covalently bound Abs, the MNPs were treated with 0.5 mM EDTA to chelate surface-bound Ni^2+^. Finally, to assess the orientation and activity of the immobilized Abs, the Trastuzumab–NTA/Dia-2@MNPs complexes were incubated with Cy3-labeled anti-human IgG (Fab specific) Ab (Cy3-anti-IgG Ab) at room temperature for 1 h. After thorough washing to remove unbound fluorophore-labeled probes, the fluorescence intensity (λ_em_ = 532 nm) was measured. Since the Cy3-anti-IgG Ab binds specifically to the Fab region of immobilized IgGs, this fluorescence readout provides a direct assessment of Ab accessibility and activity on the MNP surface.

Figure 2c illustrates how varying the surface composition of NTA and Dia-2 affects the site-specific immobilization and functional display of Ab on the MNPs. Among the five tested molar ratios, the 1:1 ratio of NTA to Dia-2 produced the highest fluorescence intensity after Cy3-anti-IgG Ab staining (Figure 2c), indicating optimal conditions for oriented and covalent Ab attachment. Although formulations with a higher proportion of NTA (e.g., 1:0 or 10:1) likely allowed more initial Ab binding, due to increased availability of Ni^2+^–NTA sites, the absence or scarcity of Dia-2 reduced the extent of covalent crosslinking. As a result, surface-bound Abs were more easily washed away, yielding lower signal intensity after the EDTA treatment. To validate that the observed signal was due to irreversible photo-crosslinking, a control experiment was performed in which UV irradiation was omitted. As shown in Figure S5, negligible fluorescence was detected following EDTA treatment in the absence of UV light, confirming that Abs bound solely through Ni^2+^–NTA coordination were removed during washing. These results underscore the importance of both Ni^2+^-mediated affinity binding and Dia-mediated covalent crosslinking for stable Ab immobilization. Based on these findings, we selected the 1:1 molar ratio of NTA/Dia-2 for all subsequent studies as it offered a balance between efficient Ab loading and effective photochemical fixation.

Next, we optimized two key parameters influencing Ab immobilization on NTA/Dia-2­(1/1)@MNPs: the Ab concentration for incubation and the UV irradiation time. To determine the maximum loading capacity, various concentrations of Trastuzumab (5–250 μg) were incubated with 1 mg of NTA/Dia-2­(1/1)@MNPs. Protein levels in both the supernatant and on the MNPs were assessed by SDS-PAGE. As shown in Figures 2d and S6, Ab loading approached saturation between 25 and 50 μg, evidenced by the appearance of unbound protein bands in the supernatant at 50 μg. To evaluate the amount of Ab covalently crosslinked to the MNPs surface, samples incubated with 5, 12.5, 25, 37.5, and 50 μg of Ab were subjected to UV irradiation (365 nm, 30 min), followed by EDTA washing to remove non-covalently bound Abs. Fluorescence assays using Cy3-anti-IgG Ab revealed that signal intensity plateaued at 25 μg Ab/mg MNPs, suggesting this concentration achieved maximal photochemical crosslinking (Figure 2d). Quantification using the BCA assay indicated an immobilization yield of approximately 11.4 μg Ab per milligram of MNPs, corresponding to ∼46% of the input Ab amount. The difference between loaded and immobilized Ab is likely due to EDTA-mediated removal of non-covalently associated Abs, consistent with the coordination-based pre-attachment mechanism. We also assessed the effect of UV irradiation time on the immobilization efficiency. Increasing exposure time from 5 to 30 min resulted in progressively higher Cy3 fluorescence, with the maximum signal achieved at 30 min (Figure 2e). Based on these findings, we selected 25 μg of Ab/mg MNPs and 30 min of UV irradiation as the optimal conditions for subsequent Ab immobilization experiments.

The morphological changes of the nanoparticles before and after Ab immobilization were further examined using TEM. As shown in Figure S7, the average diameter of NTA/Dia-2­(1/1)@MNPs was measured to be 46 ± 2 nm, confirming that the chemical derivatization with NTA and Dia-2 did not significantly alter the particle size or aggregation state. Upon Ab conjugation, the average size increased to 64 ± 4 nm, which is consistent with the addition of a monolayer of IgG Abs, whose molecular dimensions typically range from 10 to 15 nm.^38^ The Ab-conjugated MNPs also retained good aqueous dispersibility, indicating that the immobilization process preserved colloidal stability and did not induce significant particle aggregation.

### Comparison of Regioselective Ab Immobilization Methods

To evaluate the impact of the regioselective immobilization strategy, we compared the performance of NTA/Dia-2(1:1)@MNPs with that of boronic acid (BA)-functionalized MNPs (BA/Dia-1@MNPs), previously developed for glycan-targeted, oriented Ab conjugation.^31^ BAs are known to form reversible covalent complexes with *cis*-diol-containing glycans, such as those present on the *N*-glycan chain at the C_H_2 domain of the Fc region of Abs.^39,40^ Given the extensive prior characterization of boronate diester formation,^30,41^ this system serves as a well-established benchmark for evaluating regioselective Ab immobilization strategies. To prepare the BA-mediated oriented conjugates, MNPs functionalized with both BA and photoreactive Dia-1 groups (BA/Dia-1@MNPs) were synthesized following established protocols (Scheme S1).^31^ For direct comparison, MNPs with equal Ab amounts on the surface (10 μg per mg MNPs) were used, and randomly immobilized Abs (Ab-R@MNPs) on the NHS@MNPs served as control MNPs. The relative Fab-accessible activity of immobilized Abs was assessed by using the fluorescence binding assay with Cy3-anti-IgG Ab, as described previously.

As shown in Figure 3a, both NTA/Dia-2@MNPs and BA/Dia-1@MNPs, which employ site-selective, photo-crosslinking strategies, yielded significantly higher fluorescence intensities than the randomly immobilized Ab-R@MNPs. Specifically, NTA/Dia-2@MNPs exhibited a 9.2-fold increase, while BA/Dia-1@MNPs showed a 6.1-fold increase in signal compared to the randomly immobilized control. Notably, NTA/Dia-2@MNPs produced a 1.5-fold higher fluorescence intensity than BA/Dia-1@MNPs, indicating more effective exposure of the Fab region and superior preservation of Ab activity. These findings suggest that differences in immobilization topology, dictated by the specific ligand–Ab interactions, play a critical role in determining the functional orientation of surface-bound Abs. The NTA–Ni^2+^ coordination likely promotes a ‘tail-on’ orientation,^42^ as it targets the His-rich metal-binding site near the C_H_3 domain at the base of the Fc region. In contrast, boronate affinity-based immobilization, which targets the Fc *N*-glycan located near the hinge region, may result in a more variable orientation, including partial ‘flat-on’ binding.^42^ This could limit Fab accessibility and reduce the overall antigen-binding efficiency. Together, these data reinforce the conclusion that Ni^2+^–NTA-directed immobilization offers a more consistent and functionally favorable orientation for native Abs on nanoparticle surfaces. Several oriented Ab immobilization strategies have been reported in the literature, including glycan-targeted, affinity-based, and photo-crosslinking approaches, but many require Ab modification or rely on reversible interactions that limit control over Fab presentation. Our chelation-controlled approach instead uses Ni^2+^–NTA to engage the native His-rich Fc region, pre-aligning the Ab before irreversible Dia-based photo-fixation. This affinity-guided two-step covalent process enables the directional and stable immobilization of unmodified IgGs. Consistent with this design, our conjugates show higher Fab accessibility and antigen-binding activity than those of both boronate-based and randomly immobilized controls. These features highlight the practical advantages of this strategy among the current oriented immobilization methods.

**Figure 3:**
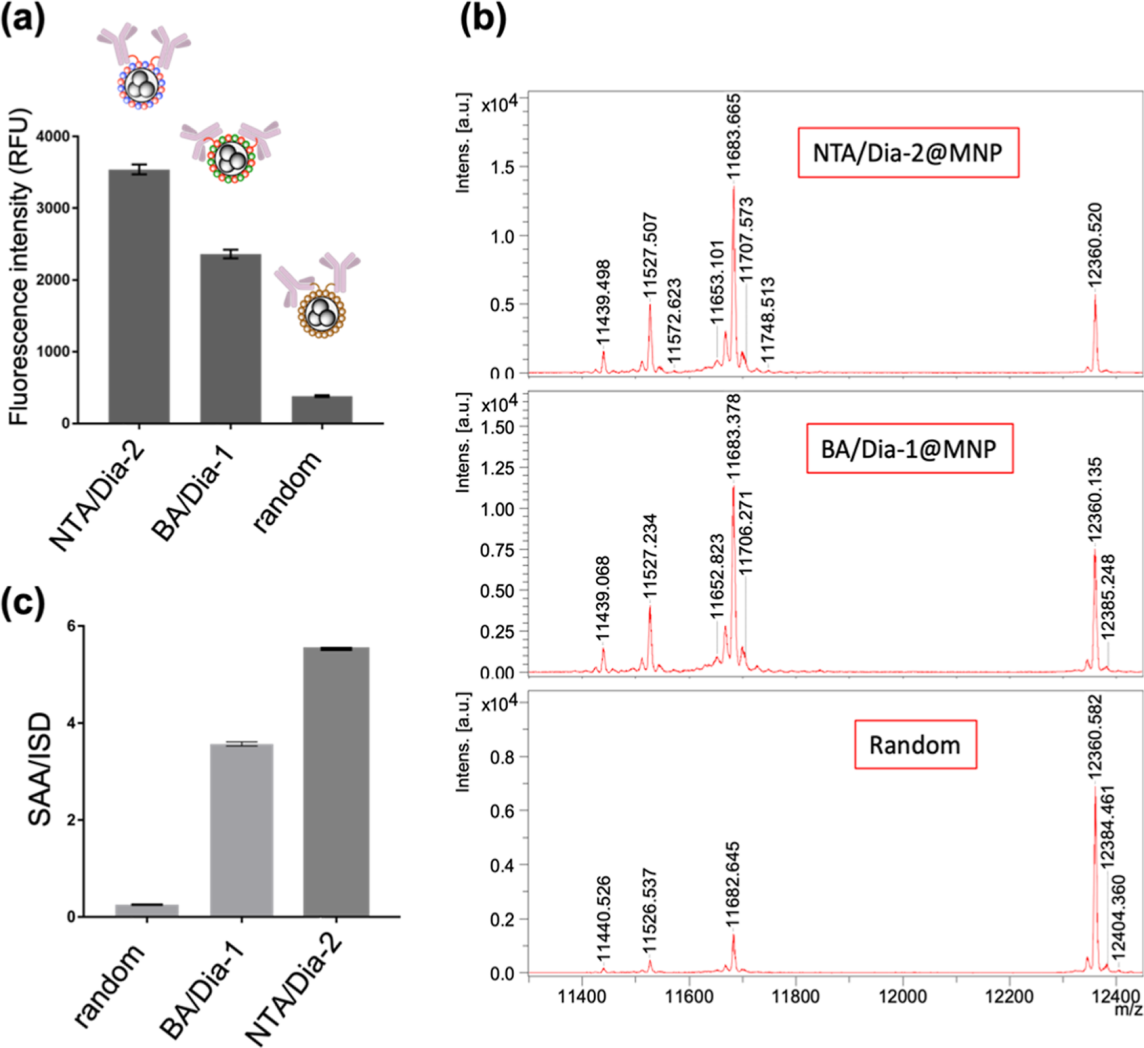
(a) Comparison of fluorescence intensity from three Ab immobilization methods: NTA/Dia-2@MNPs, BA/Dia-1@MNPs, and randomly immobilized Ab-R@MNPs, measured using Cy3-anti-IgG Ab. Extraction of SAA from human serum using different Ab−MNPs immobilization strategies. (b) MALDI-TOF mass spectra of SAA captured by anti-SAA mAb−NTA/Dia-2@MNPs (top), anti-SAA mAb–BA/Dia-1@MNPs (middle), and randomly immobilized anti-SAA mAb–R@MNPs (bottom). (c) Quantification of SAA based on the SAA/ISD signal ratio; ISD: internal standard, cytochrome c (MW: 12,360 Da). SAA variant peaks appear at ∼11,300–11,800 Da. Error bars (in a and c) represent standard deviation from triplicate experiments.

### Application of NTA/Dia-2@MNPs for Target Protein Enrichment from Cancer Patient Samples

The successful immobilization of Abs via Ni^2+^–NTA complexation on the surface of MNPs enabled us to explore their application in target protein enrichment from complex biological samples. As a proof of concept, we focused on serum amyloid A (SAA), a major acute-phase protein whose serum concentration can increase more than 1000-fold within 24 h during inflammation.^43^ In circulation, SAA predominantly forms complexes with high-density lipoproteins (HDL), and elevated levels of SAA have been reported in various cancer types, including lung, esophageal, ovarian, and gastric cancers.^44^ Therefore, quantifying SAA and analyzing its post-modification in patient serum could serve as a useful biomarker for cancer diagnosis and prognosis.^45^ Therefore, detection of predictive SAA biomarkers from patient serum samples is critical for guiding treatment decisions. To evaluate the practical utility of our site-selective immobilization strategy, we functionalized NTA/Dia-2@MNPs with an anti-SAA monoclonal Ab (anti-SAA mAb), followed by UV-induced photo-crosslinking, as described before, producing anti-SAA mAb−NTA/Dia-2@MNPs. For comparison, anti-SAA mAb was also immobilized on BA/Dia-1@MNPs and NHS@MNPs, yielding anti-SAA mAb−BA/Dia-1@MNPs and anti-SAA mAb−R@MNPs, respectively. To ensure a fair comparison, MNPs with the same amount of Ab on the surface were used. Each Ab–MNP complex was incubated with diluted serum from gastric cancer patients (5 μL serum in 55 μL of PBS) at room temperature for 1 h. Following incubation, magnetic separation was used to isolate the SAA-bound nanocomplexes. After thorough washing, the relative SAA extraction efficiency was evaluated using a nanoprobe-based affinity mass spectrometry (NBA-MS) approach.^46^ This workflow demonstrated the simplicity and efficiency of our immobilization method in real biological samples, confirming the suitability of the NTA/Dia-2@MNP system for high-specificity antigen capture and enrichment from complex media, such as human serum.

As shown in Figure 3b, by using the same amount of surface Ab, both anti-SAA mAb−NTA/Dia-2@MNPs and anti-SAA mAb−BA/Dia-1@MNPs exhibited good Ab activity for antigen enrichment, as evidenced by distinct SAA peaks (post-modification of SAA) in the MS spectra. Quantitative analysis (in comparison with internal standard, ISD, cytochrome c molecular weight 12,360 Da) of signal intensities revealed that the NTA/Dia-2-based immobilization strategy achieved 1.5-fold higher antigen capture efficiency compared to the boronate-based BA/Dia-1@MNPs system and 22-fold higher efficiency than the randomly immobilized anti-SAA mAb–R@MNPs (Figure 3c). Notably, the nonspecific protein adsorption on the MNP surfaces was minimal across all samples, indicating the high selectivity of the capture process. These results align with the findings from the fluorescence Fab-binding assay (Figure 3a), further confirming that the Fab domains remained accessible and functionally oriented on the NTA/Dia-2@MNPs surface. Moreover, the anti-SAA mAb−NTA/Dia-2@MNPs demonstrated excellent stability and functionality in the complex biological matrix of human serum, highlighting the robustness and practical utility of this immobilization approach for sensitive and selective antigen enrichment in clinical applications.

### Application in Target Protein Enrichment from Cell Lysates

To further demonstrate the superiority of the NTA–Ni^2+^-based oriented Ab immobilization, we evaluated its application in the capture of the epidermal growth factor receptor (EGFR)—a 170 kDa transmembrane glycoprotein involved in intracellular signaling pathways and one of the most widely used cancer-targeted therapies, such as colorectal carcinogenesis (CRC).^47^ As a model Ab, we selected Cetuximab, a chimeric mouse−human monoclonal Ab approved for the treatment of metastatic CRC, owing to its high specificity for EGFR and widespread clinical use.^48^ Importantly, Cetuximab is glycosylated at two distinct sites: Asn88 in the Fab domain and Asn297 in the Fc domain of the heavy chain.^49^ This dual glycosylation makes it an ideal system for evaluating how different immobilization strategies affect the Ab orientation and functionality. Using the same protocols described previously, Cetuximab was immobilized on three types of MNPs: NTA/Dia-2@MNPs, BA/Dia-1@MNPs, and NHS@MNPs, yielding Cetuximab−NTA/Dia-2@MNPs, Cetuximab−BA/Dia-1@MNPs, and Cetuximab−R@MNPs, respectively. These Ab-functionalized MNPs were applied to enrich EGFR from HEK293T cell lysates. Briefly, 0.3 mg of the total lysate protein was incubated with each type of MNP at 4 °C overnight. After magnetic separation, the captured EGFR proteins were eluted using 1% SDS and analyzed via SDS-PAGE to visualize protein enrichment (Figure S8). This setup allowed us to directly compare the antigen capture performance of each immobilization method under identical conditions.

As shown in Figures 4a and S9, by using the same amount of surface Ab, Cetuximab—NTA/Dia-2@MNPs captured a substantially greater amount of EGFR from HEK293T lysates compared to both Cetuximab−BA/Dia-1@MNPs and Cetuximab−R@MNPs. Densitometric analysis of the EGFR bands on SDS-PAGE revealed that the NTA/Dia-2-immobilized Ab achieved approximately a 6-fold increase in antigen capture relative to the other two strategies (Figure 4a). These results indicate that the Ni^2+^—NTA-mediated Fc-directed immobilization not only enhances Ab retention but also better preserves the antigen-binding activity on the MNPs surface. In contrast, the lower enrichment observed with BA/Dia-1@MNPs suggests the reduced bioactivity of the immobilized Cetuximab. Since BA chemistry targets the glycan chains of Abs, conjugation can occur at either the N297 site in the Fc domain or the N88 site in the Fab domain. Given that N88 is located near the antigen-binding epitope, immobilization via this site may sterically hinder antigen recognition, thereby compromising Ab function. Taken together, these findings underscore the importance of site-selective immobilization via the Fc domain to maintain Ab activity. The superior performance of NTA/Dia-2@MNPs in this model system highlights their general applicability for constructing functionally oriented immuno-nanoparticles.

**Figure 4:**
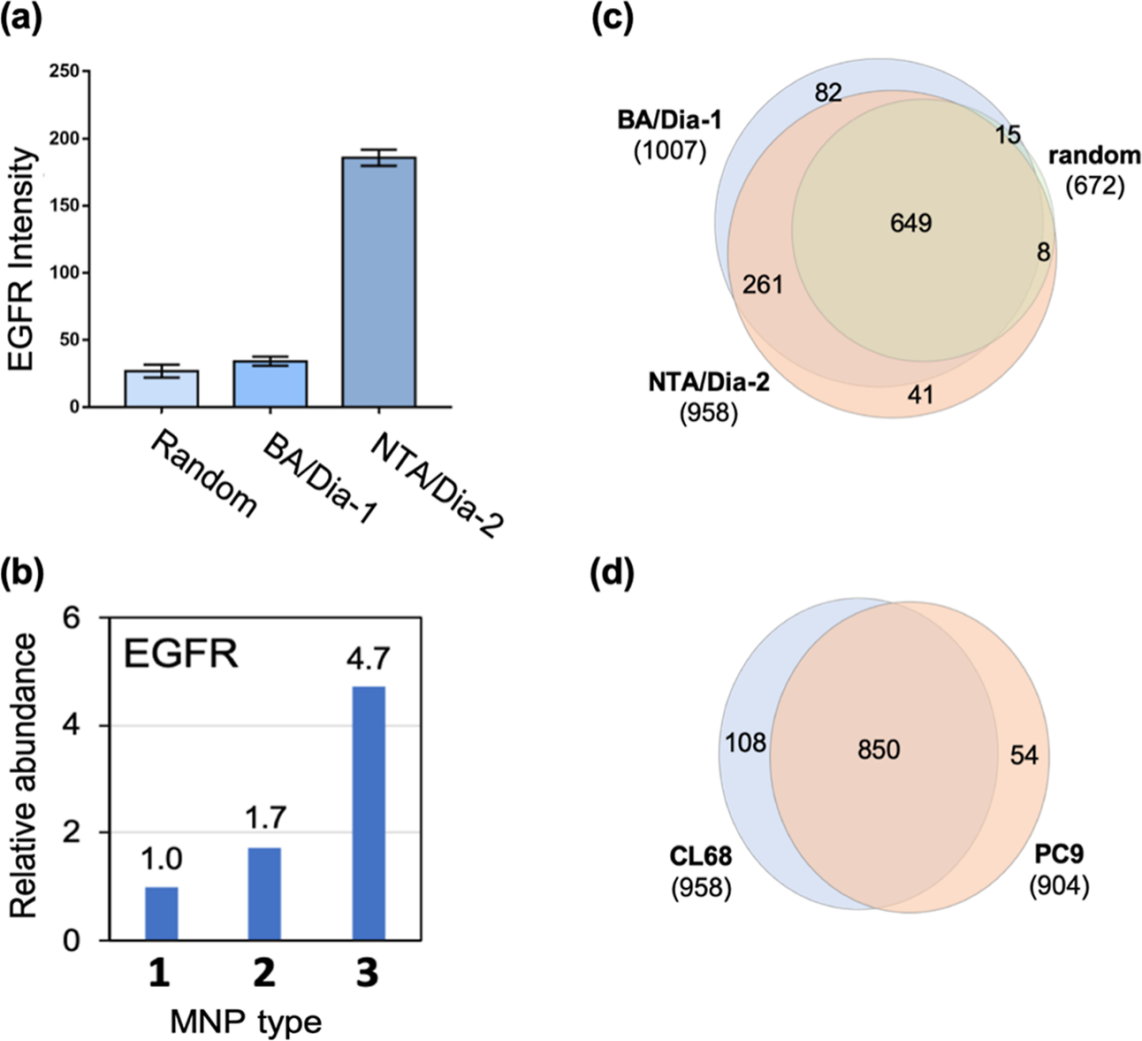
(a) EGFR enrichment from HEK293T cell lysate using Cetuximab immobilized on different MNPs. Quantification of EGFR band intensity from SDS-PAGE analysis (Figure S9) using UN-Scan-IT gel (6.1) analysis software. Experiments were performed in triplicate. GFR immunoprecipitation using three Ab–MNP conjugation strategies and analysis by LC–MS/MS. (b) Comparison of relative EGFR enrichment from the cell lysate of the NSCLC cell line (CL68) by Cetuximab–R@MNPs, BA/Dia-1@MNPs, and NTA/Dia-2@MNPs. (c) Venn diagram showing overlap among EGFR-interacting proteins captured by the three MNP platforms: 1 (random), 2 (BA/Dia-1), and 3 (NTA/Dia-2), followed by LC–MS/MS analysis for protein identification. (d) Comparative profiling of the number of common EGFR interacting proteins in NSCLC cell lines (CL68 and PC9). The Venn diagram showing the high overlap of EGFR-associated proteins identified by LC–MS/MS in the two cell lines.

To evaluate the immunoprecipitation efficiency of EGFR capture using different Ab immobilization strategies, we applied three nanoprobes, namely, Cetuximab–R@MNPs, Cetuximab–BA/Dia-1@MNPs, and Cetuximab–NTA/Dia-2@MNPs, to purify EGFR from a non-small cell lung cancer (NSCLC) cell line, CL68. The captured proteins were analyzed by LC–MS/MS in triplicate to quantify the abundance of enriched EGFR and characterize the copurified EGFR interactome, i.e. all EGFR interacting proteins. As shown in Figure 4b, the boronate-based strategy (Cetuximab–BA/Dia-1@MNPs) achieved a 1.7-fold increase compared to the randomly immobilized control (Cetuximab–R@MNPs). As a comparison, the NTA/Dia-2-based immobilization strategy exhibited the highest EGFR recovery, yielding a significantly higher (4.7-fold) increase in EGFR abundance compared to Cetuximab–R@MNPs. The results demonstrated the functional advantage of Fc-targeted orientation via NTA. Proteomic profiling further revealed higher coverage to enrich EGFR-interacting proteins by co-immunoprecipitation using Cetuximab–NTA/Dia-2@MNPs (958) and BA/Dia-1@MNPs (1007) compared to R@MNPs (672) (Figure S10). Venn diagram analysis showed that approximately 96.5% (649/672) of proteins enriched by Cetuximab–R@MNPs were also identified in the data sets from the other two MNP types. Additionally, the numbers of EGFR-interacting proteins captured by the BA- and NTA-functionalized MNPs were comparable (Figure 4c), demonstrating that both oriented strategies improved interactome coverage relative to the random immobilization method. Together, these results demonstrate that site-specific Fc-directed immobilization via NTA/Dia-2 not only significantly enhances target protein recovery but also facilitates more comprehensive analysis of protein−protein interactions of the target protein. This reinforces the value of properly oriented Ab immobilization in proteomic applications. Our results indicate that BAs can enable high Ab loading capacity on MNPs overall, especially when glycosylation is abundant and accessible, offering broad applicability across native Abs. To maximize antibody activity, NTA-based strategies provide superior control and orientation, often resulting in higher functional activity per immobilized molecule.

To further assess the general applicability of our optimized immunoprecipitation material, Cetuximab–NTA/Dia-2@MNPs were applied to explore the EGFR interactome in two NSCLC cell lines, CL68 and PC9. Using LC–MS/MS analysis, a total of 1012 EGFR-associated proteins were identified across both cell lines, with 84% (850 proteins) shared between them (Figure 4d), indicating substantial overlap in the core EGFR interactome. To gain insight into the biological significance of these protein networks, functional annotation of these identified interactome components were performed using Ingenuity Pathway Analysis (IPA). This analysis provided insights into direct and indirect protein−protein interactions as well as the regulatory architecture associated with EGFR signaling in these two mutated contexts. As shown in Figure S11, EGFR expression was modestly higher in CL68 cells compared to PC9, accompanied by upregulated abundance of PLC-gamma downstream the EGFR signaling pathway.^50^ Several key regulatory proteins also showed similar trends, including receptor-type tyrosine-protein phosphatase kappa (PTPRK), a known negative regulator of EGFR signaling; transferrin receptor protein (TFRC), a marker of cellular iron uptake and proliferation; and FUT8, which participates in glycosylation processes associated with EGFR signaling modulation.^51^ Together, these proteins highlight cell line-specific differences in EGFR-associated proteins that may contribute to differential signaling outputs and drug response. These results demonstrate the applicability of the NTA/Dia-2@MNP platform for dissecting complex signaling networks in disease-relevant models. Notably, the >1000 interacting proteins obtained in this study represent markedly greater interactome coverage compared with conventional immunoprecipitation approaches using agarose beads and the crosslinked EGFR antibody.^52^ The ability to reproducibly enrich active receptor complexes and analyze their composition by LC–MS/MS highlights the high-performance of oriented covalent Ab immobilization for interactome-scale proteomic characterization.

## Conclusions

In summary, we have developed a robust and generalizable strategy for the covalent and oriented immobilization of full-length native Abs on MNPs using a dual-functional surface composing Ni^2+^-chelated NTA and photoactivatable diazirine (Dia-2). A key advantage of this approach is that it requires no Ab engineering or chemical derivatization, enabling direct use of unmodified Abs while achieving precise Fc domain orientation via the natural His-rich cluster, thus avoiding interference with the Fab region. Subsequent photochemical locking step ensures irreversible covalent attachment, providing greater stability than the reversible boronate-diester-based method and maintaining performance under EDTA treatment and complex biological matrices. Functionally, this approach preserves high antigen-binding activity, producing up to a 9.2-fold enhancement in Fab accessibility compared to random immobilization. These advantages translate into superior biomolecular capture capabilities, including a 22-fold increase in SAA extraction over random immobilization and a 1.5-fold improvement over boronate-based orientation, as well as markedly improved immunoprecipitation performance, with a 6-fold higher EGFR enrichment and identification of more than 1000 EGFR-interacting proteins. LC–MS/MS analysis confirmed enhanced target enrichment and high interactome coverage over existing strategies, revealing the method’s utility in advanced biological applications. The simplicity, scalability, and compatibility with native Abs, without requiring Ab engineering or harsh chemical modifications, make it an attractive tool for applications in biosensing, diagnostics, proteomics, and therapeutic monitoring. Overall, this work establishes a versatile route for constructing high-performance immuno-nanomaterials and sets the stage for next-generation biofunctional surfaces.

## Supplementary Material



## Notes

Detailed experimental procedures for linker synthesis, magnetic nanoparticle (MNP) fabrication, antibody binding assays, and mass spectrometry (MS) analyses (PDF)
