# Supplementary material for: Chelation-Controlled Oriented and Irreversible Immobilization of Native Antibodies on Photoreactive Magnetic Nanoparticles
Source: ACS Appl Bio Mater. 2026 Jan 28;9(4):2118–29. doi: 10.1021/acsabm.5c02168 (PMC12914640; doi:10.1021/acsabm.5c02168)
Supplement: Supplementary file 1 [file mt5c02168_si_001.pdf]

Supporting Information

Chelation-Controlled Oriented and Irreversible Immobilization of Native Antibodies on Photoreactive Magnetic Nanoparticles

Yi-Ren Huo,[a] *Avijit K. Adak,*[a,*] *Sachin K. Kawade,*[a] *Yi-Ju Chen,*[b] *Mira Anne C. dela Rosa,*[c] *Yu-Ju Chen,*[b,*] *and Chun-Cheng Lin*[a,d,*]

[a]Department of Chemistry, National Tsing Hua University, Hsinchu 300044, Taiwan

[b]Institute of Chemistry, Academia Sinica, Taipei 115201, Taiwan

[c]JunZhi Biomedical Co., Ltd., Tainan 744094, Taiwan

[d]Department of Medicinal and Applied Chemistry, Kaohsiung Medical University, Kaohsiung 807378, Taiwan

Corresponding author's E-mail: cclin66@mx.nthu.edu.tw

Table of content **Page No.**

1. The workflow of BA/Dia-1 and random immobilization (**Scheme S1**): S3
2. The TEM spectra of (a) Fe_3O_4 core, (b) NH_2@MNPs, (c) NTA@MNPs, (d) Dia-1@MNPs, (e) Dia-2@MNPs (**Figure S1**): S4
3. The SDS-PAGE of EDTA competition with Trastuzumab-NTA@MNPs (**Figure S2**): S5
4. The screening test of (a) incubation time and (b) pH range (**Figure S3**): S5
5. Trastuzumab photo-immobilization on Dia-1@MNP in the presence of Cu^{2+}(**Figure S4**): S5
6. Fluorescence intensity of Cy3-anti-IgG Ab binding to Trastuzumab immobilized on NTA/Dia-2@MNPs with varying NTA/Dia-2 ratios without UV irradiation (**Figure S5**): S6
7. The maximum antibody payload before UV irradiation (**Figure S6**): S6
8. The TEM spectra of (a) NTA/Dia-2(1/1)@MNP and (b) Trastuzumab-NTA/Dia-2(1/1)@MNPs (**Figure S7**): S7
9. Schematic workflow of EGFR extraction (**Figure S8**): S7
10. EGFR enrichment from HEK293T cell lysate using Cetuximab immobilized on different MNPs (**Figure S9**): S7
11. Number of EGFR-interacting proteins identified by each method (**Figure S10**): S8
12. Protein–protein interaction networks and regulatory pathways of the EGFR interactome analyzed using Ingenuity Pathway Analysis (IPA) (**Figure S11**): S8
13. General materials, methods, and instrument: S9
14. References: S10

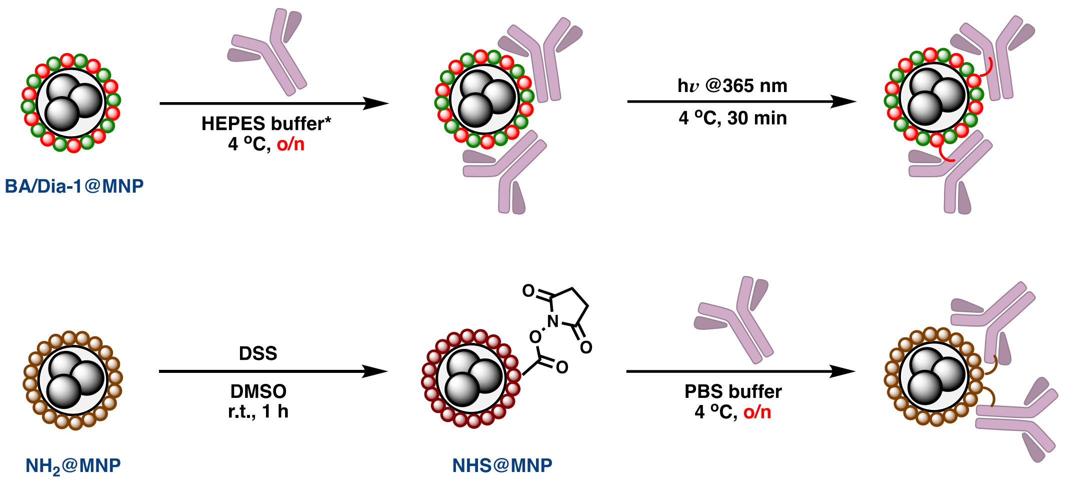

Scheme S1. The workflow of BA/Dia-1 and random immobilization.

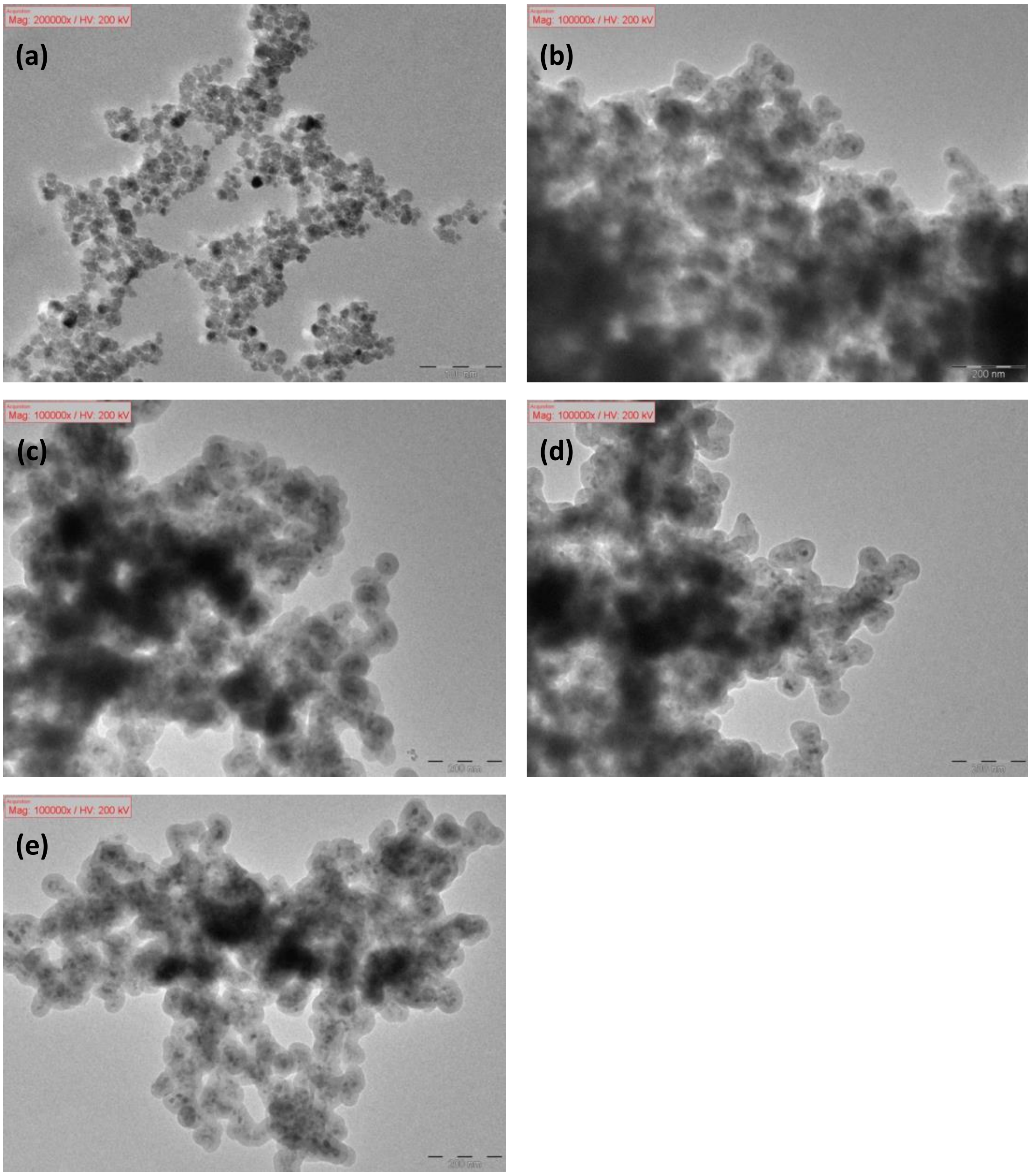

Figure S1. The TEM spectra of (a) Fe_3O_4 core, (b) NH_2@MNPs, (c) NTA@MNPs, (d) Dia-1@MNPs, (e) Dia-2@MNPs.

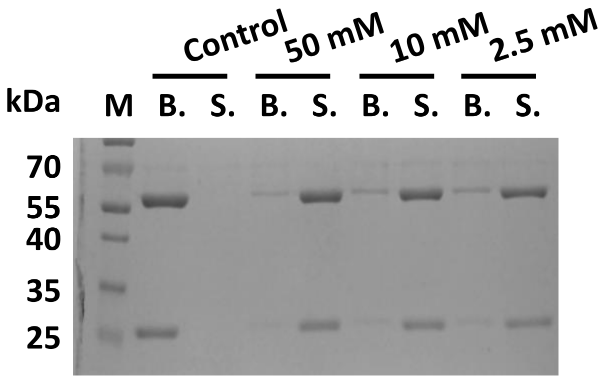

Figure S2. The SDS-PAGE of EDTA competition with Trastuzumab-NTA@MNPs. The resulting MNP precipitate was added 2.5, 10, 50 mM EDTA in PBS buffer respectively. The resulting mixture was shaken vigorously at 4 °C for 10 min.

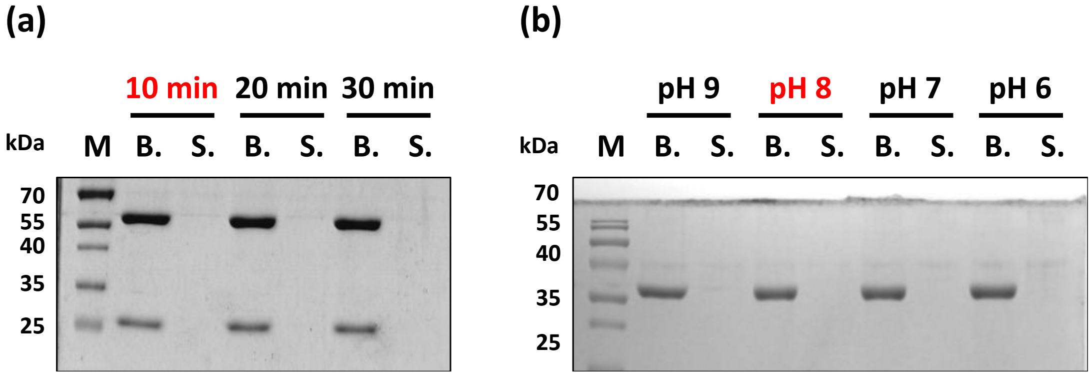

Figure S3. The screening test of (a) incubation time and (b) pH range.

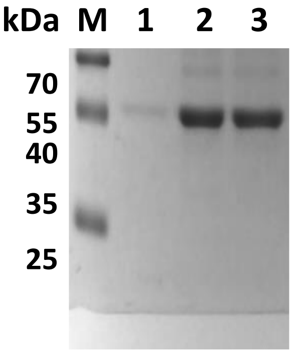

Figure S4. Trastuzumab photo-immobilization on Dia-1@MNP in the presence of Cu^{2+}. Lane 1: the supernatant before irradiation. Lane 2: the MNPs before irradiation. Lane 3: the MNPs after irradiation.

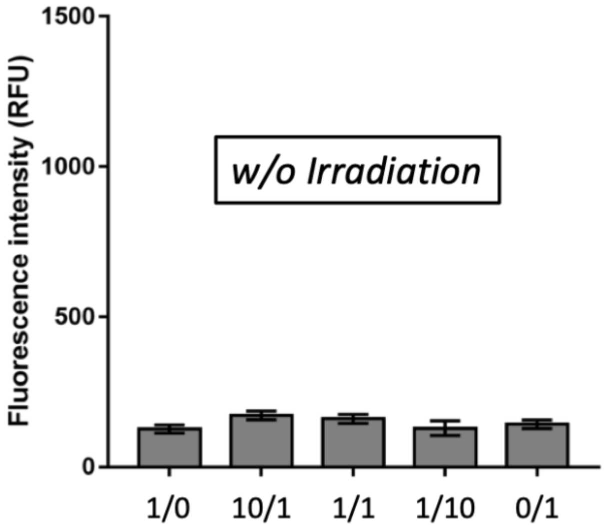

Figure S5. Fluorescence intensity of Cy3-anti-IgG Ab binding to Trastuzumab immobilized on NTA/Dia-2@MNPs with varying NTA/Dia-2 ratios without UV irradiation. Error bars represent standard deviation from triplicate experiments.

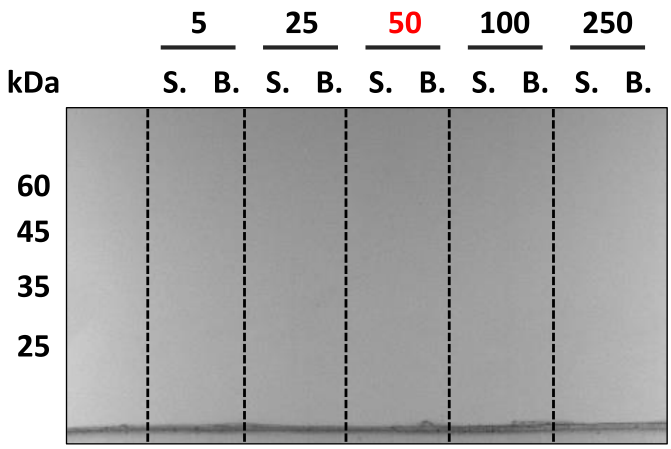

Figure S6. The maximum antibody payload before UV irradiation. From SDS-PAGE, when antibody/MNP ratio is 50 ug Ab/mg MNP, NTA/Dia-2(1/1)@MNP will reach maximum payload (non-covalent).

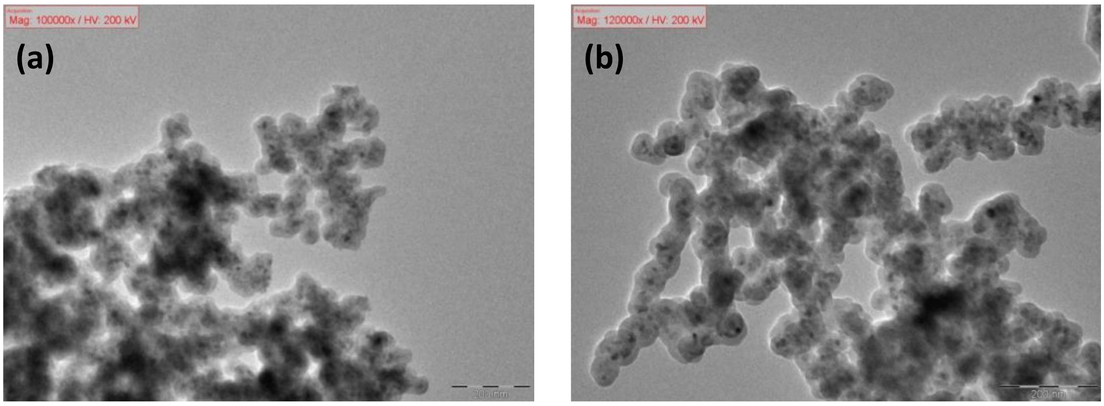

Figure S7. The TEM spectra of (a) NTA/Dia-2(1/1)@MNP and (b) Trastuzumab-NTA/Dia-2(1/1)@MNPs.

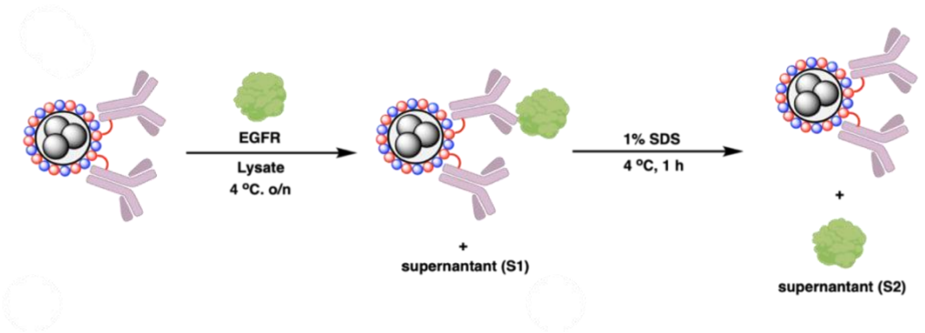

Figure S8. Schematic workflow of EGFR extraction. EGFR enrichment from HEK293T cell lysate using Cetuximab immobilized on different MNPs.

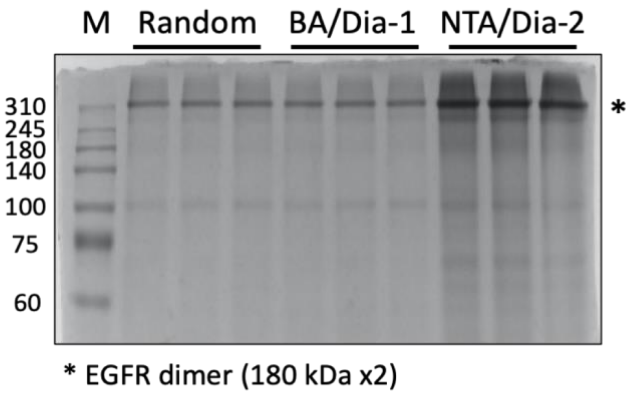

Figure S9. EGFR enrichment from HEK293T cell lysate using Cetuximab immobilized on different MNPs. SDS-PAGE analysis of supernatants after 1% SDS elution (S2); the asterisk (*) denotes EGFR dimer.

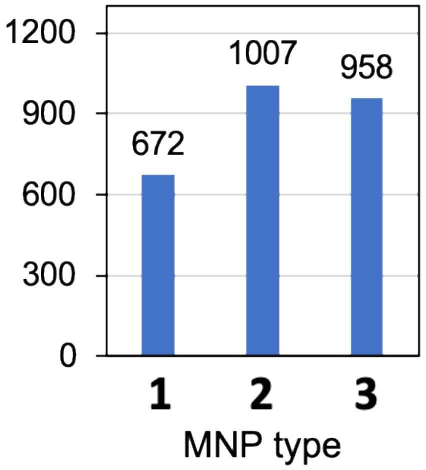

Figure S10. Number of EGFR-interacting proteins identified by each method. MNP type: **1** (random), **2** (BA/Dia-1), **3** (NTA/Dia-2).

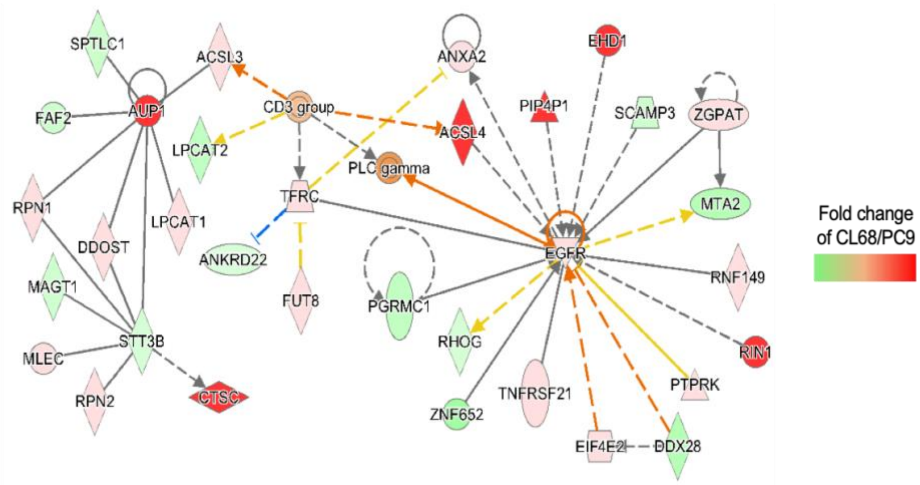

Figure S11. Protein–protein interaction networks and regulatory pathways of the EGFR interactome analyzed using Ingenuity Pathway Analysis (IPA).

General material and instrument

The chemicals for the synthesis and biological reagents were all obtained from Acros, Merck, Fluka, Tokyo Chemical Industry (TCI), or Sigma-Aldrich and used without further purification unless otherwise notice. Trastuzumab was purchased from Roche (Herceptin). Anti-SAA mAb was purchased from Anogen (MO-C40028A). Cetuximab was purchased from Selleckchem (A2000). anti-human IgG (Fab' specific) antibody-Cy3 was purchased from Jackson Immunoresearch (#109-165-170). Photoirradiation experiment was performed under 365 nm by Blak-Ray® B-100AP High Intensity UV lamp. Sodium dodecyl sulfate-polyacrylamide gel electrophoresis (SDS-PAGE) were carried out with Bio-Rad Mini-Protean III electrophoresis apparatus. The fluorescence signals were collected by fluorometer (Tecan, Infinite M200 pro). The TEM figure of MNPs were measured by transmission electron microscope (JEM2000 FXII) at NCU.

General protein analysis procedure

The antibody solution in SDS-PAGE loading dye (0.2% bromophenol blue, 0.1 M Tris-HCl, 4% SDS, 20% glycerol, pH 6.8) was the treated with 3% DTT and then was heated to 95 °C for 10 min before being analyzed by electrophoresis. The protein signal was visualized by Coomassie blue staining. The samples for native PAGE were untreated with DTT and without boiling process.

Synthesis of NTA, Dia-1, and Dia-2

NTA,[1] Diaz-1,[2] and Dia-2[3] were prepared and characterized according to literature procedures.

References

1. Zheng, Y.; Wegner, T.; Iorio, D. D.; Peirau, M.; Glorius, F.; Wegner, S. V. NTA-Cholesterol analogs for the nongenic liquid-ordered phase-specific functionalization of lipid membranes with proteins. *ACS Chem. Biol.* **2023**, *18*, 1435-1443.

2. Fan, C.-Y.; Hou, Y.-R.; Adak, A. K.; Waniwan, J. T.; dela Rosa, M. A. C.; Low, P. Y.; Angata, T.; Hwang, K.-C.; Chen, Y.-J.; Lin, C.-C., Boronate affinity-based photoactivatable magnetic nanoparticles for the oriented and irreversible conjugation of Fc-fused lectins and antibodies. *Chem. Sci.,* **2019**, *10*, 8600-8609.

3. Adak, A. K.; Li, B.-Y.; Huang, L.-D.; Lin, T.-W.; Chang, T.-C. Hwang, K. C.; Lin, C.-C. Fabrication of antibody microarrays by light-induced covalent and oriented immobilization. *ACS Appl. Mater. Interfaces* **2014**, *6*, 10452−10460.
